# Giant Cell Hepatitis – A Rare Association with Connective Tissue Disease

**DOI:** 10.31138/mjr.30.4.224

**Published:** 2019-12-31

**Authors:** Maria Rauf, Sambit Sen, Adam Levene, Muhammad K. Nisar

**Affiliations:** 1 Histopathology Department, Luton & Dunstable University Hospital, Luton, United Kingdom,; 2 Hepatology Department, Luton & Dunstable University Hospital, Luton, United Kingdom,; 3 Rheumatology Department, Luton & Dunstable University Hospital, Luton, United Kingdom

**Keywords:** giant cell hepatitis, connective tissue disease, antiphospholipid antibodies

## Abstract

A 68-year-old gentleman presented to hepatology department with asymptomatic year-long history of stably deranged liver function tests. His peak alkaline phosphatase (ALP), was 828 with alanine transaminase (ALT) of 141. Full liver workup was negative; hence, a liver biopsy was organised, which confirmed giant cell hepatitis (GCH). A computed tomography (CT) scan revealed non-specific interstitial pneumonitis (NSIP) pattern interstitial lung disease supported by lung function tests. Antibody testing showed strongly positive antinuclear antibody (ANA) with anti-polymyositis/scleroderma (anti-PM-SCL) antibody. Clinical picture was in keeping with likely undifferentiated connective tissue disease (UCTD) with polyarthralgia, early morning stiffness, Raynaud’s and nailfold infarcts with capillaritis on nail bed examination. Further testing confirmed triple-positive antiphospholipid antibodies twice 12 weeks apart (immunoglobulin M [IgM] anti beta-2 glycoprotein antibodies, lupus anticoagulant and IgM anticardiolipin antibody). He was treated with mycophenolate and hydroxychloroquine with resolution of symptoms. Giant cell hepatitis is uncommon, with only 100 cases reported worldwide. To our knowledge, this is the only report of GCH in the context of UCTD, highlighting the significance of careful evaluation of liver disease overlap and the successful role of mycophenolate mofetil (MMF) in this setting.

## INTRODUCTION

Giant cell hepatitis (GCH) is a condition characterized by inflammation and large multinucleated hepatocytes in the hepatic parenchyma *( Figure 1A–C)*.^1^ The condition is heterogeneous and clinical presentation depends on underlying aetiology. This could vary from mild hepatitis to liver cirrhosis and fulminant liver failure.^2^ Infections and drugs have been described as predominant triggers, with fewer reports in the context of autoimmune disease.^3^ Considering that rheumatologists are primarily involved in the management of patients with autoimmune rheumatic diseases, it is imperative to be aware of potential overlap between GCH and such conditions. We present a case of a man with giant cell hepatitis, interstitial lung disease and undifferentiated connective tissue disorder with triple positive antiphospholipid antibodies.

**Figure 1a.**
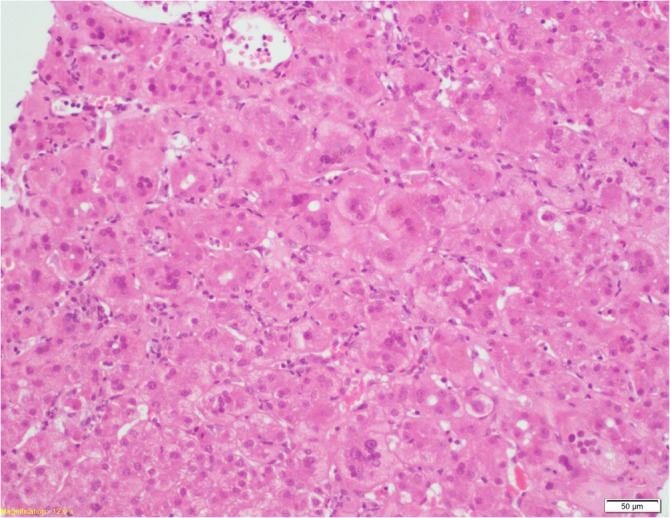
×200 giant cell multinucleated hepatocytes, focal glassy eosinophilic cytoplasm, lobular inflammation.

**Figure 1b.**
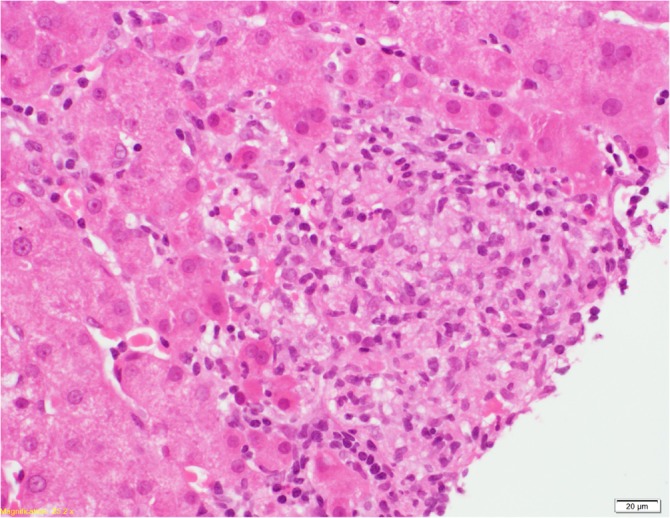
×400 area of vague non-necrotising granulomatous inflammation.

**Figure 1c.**
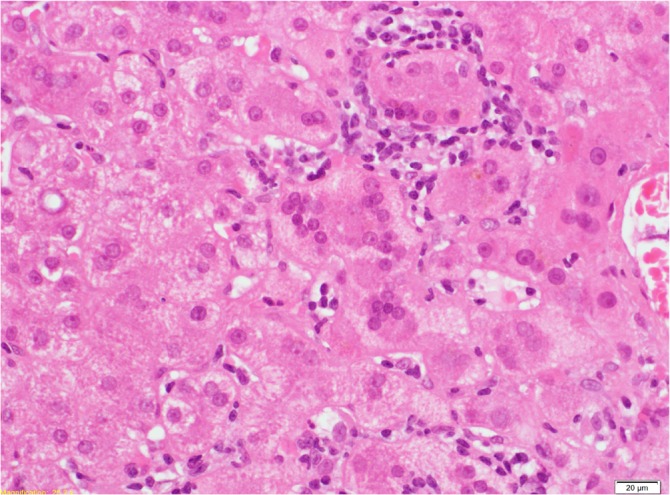
×400 area of vague non-necrotising granulomatous inflammation.

## CASE DESCRIPTION

A 68-year-old gentleman, with limited mobility owing to multiple sclerosis-related spastic paraparesis for 15 years, presented to hepatology department with asymptomatic year-long history of stably deranged liver function tests. Examination was unremarkable with lack of liver disease signs and symptoms (no evidence of portal hypertension, palmar erythema, ascites or spider naevi). His peak alkaline phosphatase (ALP) was 828 with alanine transaminase (ALT) of 141. He underwent a range of investigations including ultrasound, triple-phase computed tomography (CT) scan of the liver, magnetic resonance cholangiopancreatography (MRCP), liver antibodies and viral screen including hepatitis B, C and HIV which were all unremarkable. Hence, the patient was consented for liver biopsy, which confirmed GCH – an unusual finding in an adult. Consequently, he underwent further screening including Epstein-Barr virus (EBV), cytomegalovirus (CMV), Hep A and E, and parvovirus PCR and serology testing, which were all negative.

In order to exclude an occult neoplasm, a CT scan of thorax, abdomen and pelvis was organised, which incidentally revealed non-specific interstitial pneumonitis (NSIP) pattern interstitial lung disease. His lung function tests showed restrictive pattern with low transfer factor. Echocardiogram showed post-capillary pulmonary hypertension with PA pressure of 38–40mm of Hg. As his mobility was limited, he was not particularly dyspnoeic however he did report persistent dry cough. Antibody testing showed strongly positive antinuclear antibody (ANA) (1:1000 by Hep 2 cells) in homogeneous pattern with anti-polymyositis/scleroderma (PM-SCL) antibody; hence, he was referred to our unit. Clinical picture was in keeping with likely undifferentiated connective tissue disease with polyarthralgia (no synovitis), early morning stiffness, Raynaud’s and nailfold infarcts with capillaritis on nail bed examination. In view of latter findings, further testing was undertaken which confirmed triple positive antiphospholipid antibodies twice 12 weeks apart (IgM anti beta-2 glycoprotein antibodies, lupus anticoagulant and IgM anticardiolipin antibody). His erythrocyte sedimentation rate (ESR) was also elevated at 46mm/hr. Rest of the autoimmune screen was negative. Renal function was persistently normal. He never had any thromboembolic events, and no blood dyscrasias.

In view of multisystem involvement with rheumatic symptoms, hydroxychloroquine 200mg twice daily was commenced. There was no improvement demonstrated at three months’ review. Following an MDT discussion with hepatologist and respiratory physician, mycopheno-late mofetil (MMF) was initiated with gradual uptitration to 15mg/kg/day. Within six weeks, good improvement was noticed with resolution of nail-fold infarcts and arthralgias. ESR dropped to 30mm/hr. Both ALP and ALT improved to 384 and 71 respectively (*Table 1*). A year later he remains well with no new symptoms. His cough and high-resolution computed tomography (HRCT) scan of chest improved as well.

**Table 1.** Biochemical workup.

**Test**	**At presentation**	**Pre-MMF**	**Post-MMF**	**Normal value**
**LFTs**
**Bilirubin**	7	9	8	2–20 μmol/L
**Albumin**	37	36	40	35–50 g/L
**ALT**	101	141	71	5–30 U/L
**ALP**	457	828	384	50–100 U/L
**AST**	97	103	69	5–30 U/L
**GGT**	187	203	111	6–50 U/L
**INR**	0.9	0.9	1.0	0.9–1.2

ALP: alkaline phosphatase; ALT: alanine transaminase; LFTs: liver function tests; AST: aspartate aminotransferase; GGT: gamma-glutamyl transpeptidase; INR: international normalized ratio.

## DISCUSSION OF SIMILAR PUBLISHED CASES

To our knowledge, this is the only report of three apparently different but overlapping diagnoses in a single patient. GCH is highly uncommon in adults.^4^ Latest review found only 100 cases reported worldwide. Up to 40% of these had an association with autoimmune disease, with autoimmune hepatitis being the most common.^3^

There are no reports of antiphospholipid antibodies or interstitial lung disease in this context.

Common causes for GCH are drugs exposure with methotrexate and 6-mercaptopurine implicated in few reports. In such cases, outcomes have been largely positive after cessation of offending medication.^5^ Viral infections, on the other hand, have had serious consequences with higher mortality.^6^ Concurrent neoplasms have occasionally been described including chronic leukaemias.^7^ A range of autoimmune disorders have been associated with GCH including rheumatoid arthritis, connective tissue diseases, inflammatory bowel disease and vasculitis.^8^ In such scenarios, ANA is a common finding, although mechanism of giant cell development is unknown. Clinical course is variable, and is dependent on the severity of liver disease at diagnosis with most cases developing rapidly progressive cirrhosis.

Common treatments employed for GCH with associated autoimmune rheumatic diseases tend to have reasonable prognosis when treated with corticosteroids or azathioprine.^9^ MMF has not been previously used in this scenario; however, it is a well-established immunomodulator used to treat several autoimmune diseases. It reversibly inhibits the enzyme pivotal in purine synthesis, thereby restraining the proliferation of B and T cells. Hence, in addition to its established place in transplant medicine, the role of MMF now spans various specialities and is increasingly utilised to treat multisystem conditions.^10^ Certainly, the role of MMF in lupus and indeed lupus nephritis is well established. Similarly, it has been increasingly used in antiphospholipid antibody positive disease complex. There is growing evidence of its benefits in connective tissue diseases. We chose it for its broad impact on suppressing both T and B cell lineages which are implicated in all the concerned disease processes in our patient.

Abnormal liver function tests (LFTs) are quite common in rheumatology practice. Such minor aberrations tend to be related to immunomodulators or steatosis. In this case, though major improvement noted, LFTs remain abnormal despite treatment suggestive of mild liver disease. However, reassurance is drawn from full liver workup and the security of diagnosis. Our case underscores the significance of careful evaluation of a challenging disease overlap with a need to investigate the cause of abnormal LFTs and the successful role of MMF in this setting.

## ABBREVIATIONS

ALP: Alkaline phosphatase
ALT: Alanine transaminase
ANA: Antinuclear antibody
CMV: Cytomegalovirus
CT: Computed tomography
EBV: Epstein-Barr virus
ESR: Erythrocyte sedimentation rate
GCH: Giant cell hepatitis
HRCT: High-resolution computed tomography
IgM: Immunoglobulin M
LFTs: Liver function tests
MRCP: Magnetic resonance cholangiopancreatography
MMF: Mycophenolate mofetil
NSIP: Non-specific interstitial pneumonitis
PM-SCL: Polymyositis/scleroderma
UCTD: Undifferentiated connective tissue disease

## References

[1] Lau JYN Koukoulis G Mieli-Vergani G Portmann BC Williams R. Syncytial giant-cell hepatitis—a specific disease entity? J Hepatol 1992;15(1–2):216–9. [10.1016/0168-8278(92)90039-r] [PMID: ] 1506641

[2] Gábor L Pál K Zsuzsa S. Giant cell hepatitis in adults. Pathol Oncol Res 1997;3(3):215–8. [10.1007/BF02899924] [PMID: ] 18470733

[3] Bihari C Rastogi A Sarin SK. Postinfantile giant cell hepatitis: an etiological and prognostic perspective. Hepat Res Treat 2013;2013:601290. [10.1155/2013/601290] [PMID: ] [PMCID: ] 23555054 PMC3608114

[4] Johnson SJ Mathew J MacSween RNM Bennett MK Burt AD. Post-infantile giant cell hepatitis: histological and immunohistochemical study. J Clin Pathol 1994;47(11):1022–7. [10.1136/jcp.47.11.1022] [PMID: ] [PMCID: ] 7829677 PMC503066

[5] Tordjmann T Grimbert S Genestie C Freymuth F Guettier C Callard P [Adult multi-nuclear cell hepatitis. A study in 17 patients]. Gastroenterol Clin Biol 1998;22(3):305–10. [PMID: ] 9762216

[6] Micchelli STL Thomas D Boitnott JK Torbenson M. Hepatic giant cells in hepatitis C virus (HCV) mono-infection and HCV/HIV co-infection. J Clin Pathol 2008;61(9):1058–61. [10.1136/jcp.2008.058560] [PMID: ] 18682418

[7] Gupta E Yacoub M Higgins M Al-Katib AM. Syncytial giant cell hepatitis associated with chronic lymphocytic leukemia: a case report. BMC Blood Disord 2012 7 19;12:8. [10.1186/1471-2326-12-8] [PMID: ] [PMCID: ] 22812631 PMC3502519

[8] Dohmen K Ohtsuka S Nakamura H Arase K Yokogawa Y Asayama R Post-infantile giant cell hepatitis in an elderly female patient with systemic lupus erythematosus. J Gastroenterol 1994;29(3):362–8. [10.1007/BF02358378] 8061807

[9] Tajiri K Shimizu Y Tokimitsu Y Tsuneyama K Sugiyama T. An elderly man with syncytial giant cell hepatitis successfully treated by immunosuppressants. Intern Med 2012;51(16):2141–4. [10.2169/internalmedicine.51.7870] [PMID: ] 22892492

[10] Van Gelder T Hesselink DA. Mycophenolate revisited. Transpl Int 2015;5;28(5):508–15. [10.1111/tri.12554] [PMID: ] 25758949

